# Comparison of BMI Derived from Parent-Reported Height and Weight with Measured Values: Results from the German KiGGS Study

**DOI:** 10.3390/ijerph9020632

**Published:** 2012-02-16

**Authors:** Anna-Kristin Brettschneider, Ute Ellert, Angelika Schaffrath Rosario

**Affiliations:** Department of Epidemiology and Health Reporting, Robert Koch Institute, 13302 Berlin, Germany; Email: EllertU@rki.de (U.E.); RosarioA@rki.de (A.S.R.)

**Keywords:** children and adolescents, parental reports, height, weight, BMI, overweight

## Abstract

The use of parent-reported height and weight is a cost-efficient instrument to assess the prevalence of children’s weight status in large-scale surveys. This study aimed to examine the accuracy of BMI derived from parent-reported height and weight and to identify potential predictors of the validity of BMI derived from parent-reported data. A subsample of children aged 2–17 years (n = 9,187) was taken from the 2003–2006 cross-sectional German KiGGS study. Parent-reported and measured height and weight were collected and BMI was calculated. Besides descriptive analysis, linear regression models with BMI difference and logistic regression models with weight status misclassification as dependent variables were calculated. Height differences varied by gender and were generally small. Weight and BMI were under-reported in all age groups, the under-reporting getting stronger with increasing age. Overall, the proportion for overweight and obesity based on parental and measured reports differed slightly. In the youngest age group, the proportion of overweight children was overestimated, while it was underestimated for older children and adolescents. Main predictors of the difference between parent reported and measured values were age, gender, weight status and parents’ perception of the child’s weight. In summary, the exclusive use of uncorrected parental reports for assessment of prevalence rates of weight status is not recommended.

## 1. Introduction

Globally, obesity has become increasingly more prevalent in children of all ages [1], though the increase is possibly coming to a standstill [2,3,4]. The German Health Interview and Examination Survey for Children and Adolescents (KiGGS) showed that the proportion of overweight and obese children and adolescents aged 3–17 years has increased about 50% compared to the 1990s [5]. Therefore an accurate monitoring of body mass index (BMI) is important for the diagnosis, prevention and reduction of overweight and obesity. Self-reported or proxy-reported height and weight, assessed with self-administered questionnaires or telephone interviews, are an attractive and more cost-efficient option compared to measuring and are therefore getting more popular in large-scale studies.

Previous studies report that both adults and adolescents tend to over-report their own height and underestimate their weight, with the consequence of an understated BMI [6,7,8,9,10,11]. The same has been found for adolescent self-reports in the KiGGS population [12]. However, the picture regarding the accuracy of parental reports of their children’s height and weight is more complex. Results of previous studies for height were mixed. Huybrechts *et al.* [13], who examined parents’ reports of 3–7-year-olds from Belgium, and Wing *et al.* [14], who analyzed parental information from British children aged 6–12 years, found an overestimation of height. But the study of Davis and Gergen [15], who examined mothers’ reports of Mexican American children aged 6 months to 11 years, found an underestimation of height. The same holds true for the study of 2–17-year-olds by Akinbami and Ogden in the U.S., except for the group of 16–17 year old adolescents, where height was estimated almost exactly. Weight was underestimated through parental reports [16], except for the group of 2–3-year old adolescents, where height was estimated almost exactly. However, this study compared measured data from a nationally representative U.S. examination survey with parent-reported data from another nationally representative survey, which limits the comparability. Weight was underestimated through parental reports [13,14,15,16], except for the group of 2–3-year-olds in the study of Akinbami and Ogden [16]. For this group weight was overestimated. The results for BMI were contradictory. Huybrechts *et al.* [13] found an underestimated BMI derived from parental reports which led to a downward bias in the prevalence rates for overweight. Akinbami and Ogden [16] found an overestimation of the prevalence rates for overweight based on reported data in younger children, getting weaker with increasing age up to an underestimation for adolescents from the age of 12 years onwards. Parents of underweight children and adolescents tended to overestimate their children’s weight status based on reported data while parents of overweight children were inclined to under-report their children’s weight status [15,17].

The purpose of the present study was first to evaluate the accuracy of BMI derived from parent-reported height and weight in a representative sample of 2–17-year old children and adolescents, using individual differences in BMI derived from parent-reported *vs.* measured height and weight. The second objective was to identify potential predictors of the validity of BMI derived from parental reports.

## 2. Experimental Section

### 2.1. Study Population

In this study the data of a subset of the German Health Interview and Examination Survey for Children and Adolescents (KiGGS) is analyzed. From May 2003 to May 2006 a total of 17,641 boys and girls aged 0–17 years, from 167 study locations representative for Germany, were surveyed (response rate 66.6%). Participants beyond 14 years of age and all parents or caregivers provided written informed consent prior to the interview and examination. The survey was approved by the Federal Office for Data Protection and by the ethics committee of Charité University Medicine Berlin. Amongst others, self-administered questionnaires filled in by parents, and physical examinations were included in the survey [18]. Parental reports of their children’s height and weight were only collected in the second half of the survey (starting in August 2004). Participants with implausible (n = 5) or missing values for measured or parent-reported height and weight were excluded. Measured height data was missing or implausible for 33 (0.3%) and measured weight data for 62 children and adolescents. Subsequently, for 78 children (0.8%) BMI based on measurements could not be calculated. Parent-reported height was missing or implausible for 532 (5.4%) and parent-reported weight for 502 (5.1%) individuals, which led to 599 (6.1%) missing values for BMI calculated from self-reports. In total, for 670 (6.8%) individuals BMI difference between self-reported and measured values could not be calculated. Because of the limited validity of the national German BMI reference [19] for children under 2 years of age [20], the sample is restricted to children and adolescents aged 2 to 17 years (n = 9,187).

### 2.2. Anthropometric Data and Parental Reports

Anthropometric measurements were taken by trained staff using standardized methods. Body height was measured, without wearing shoes, with an accuracy of 0.1 cm, using a portable Harpenden stadiometer (Holtain Ltd., UK). Body weight was measured to the nearest 0.1 kg, wearing underwear, with a calibrated electronic scale (SECA, Ltd., Germany). Immediately prior to the standardized measurement, parents were asked face-to-face to report height and weight of their children with an accuracy of 1 cm or 1 kg, respectively. Information about the study procedure was available in the internet beforehand.

Body mass index (BMI) in kg/m² was calculated from parent-reported and from measured data. Weight status was classified according to age and gender into underweight (<10th percentile), normal weight (≥10th percentile to ≤90th percentile) and overweight (>90th percentile) based on the national German reference [19]. Throughout this paper, the category ‘overweight’ includes obese children.

### 2.3. Potential Predictors of the Quality of Parent-Reported Height, Weight and BMI

Parental perception of their child’s weight was examined by asking the following question in the self-administered questionnaire: ‘In your view, is your child…’ ‘far too thin’, ‘slightly too thin’, ‘exactly the right weight’, ‘slightly too fat’, or ‘far too fat’ [21]? Responses were classified into the following categories: (1) ‘too thin’ (summarizes ‘far too thin’ and ‘slightly too thin’); (2) ‘right weight’ (equivalent to ‘exactly the right weight’); and (3) ‘too fat’ (summarizes ‘slightly too fat’ and ‘far too fat’).

Data on parents’ income, occupational status, and educational and occupational qualification from the parental questionnaire were used to quantify the socio-economic status of the children and adolescents. Each of the three components was rated with a point system (1–7 points). The sum was calculated and categorised into the following groups: (1) low (3–8 points); (2) medium (9–14 points); and (3) high (15–21 points) socio-economic status [22]. Participants were referred to as immigrants if they had immigrated themselves and had at least one parent who was not born in Germany or was of non-German nationality, or if both parents had immigrated or were of non-German nationality [23]. Self-reported height and weight of mothers and fathers were used to calculate parental BMI which was classified into overweight (yes/no) according to the WHO cut-off point of ≥25 kg/m^2^ [1]. They were allocated to the following categories: (1) both parents overweight; (2) one parent overweight (including overweight single parents); and (3) no parent overweight.

### 2.4. Statistical Analysis

SPSS 14 for Windows (Chicago, IL, USA) was used for data management and statistical calculations. The study population was examined according to age groups (2–6, 7–10 and 11–17 years) and sex. Overall as well as gender- and age group-specific proportions for the different categories of weight status were calculated. The difference in the proportion of overweight based on parent-reported *vs.* measured height and weight was assessed with the McNemar test for paired data. Sensitivity and specificity for underweight and overweight were assessed and expressed as percentages [24]. Confidence intervals (CI) for sensitivity and specificity were calculated via the normal approximation for binomial proportions. 

The individual differences between parent-reported and measured values for height, weight and BMI derived thereof were calculated as parental report minus measured value, so that positive differences indicate overestimation of the measured value by the parental reports. Mean differences were tested for difference from zero with the paired samples t-test. Mean differences between boys and girls and between the age groups were tested with Student’s t-test and F-test, respectively. 

The difference between BMI based on parent-reported *vs.* measured height and weight was examined in linear regression models (Model 1) which were adjusted for age (in years, as a categorical variable). The effect of potential predictors of the validity of parental reports on the difference was analyzed separately for boys and girls. In order to develop a multiple linear regression model, all potential predictors were initially screened in simple linear regression analyses, including only one predictor at a time (plus age as an adjustment variable). Resulting from this procedure all variables which showed significance (*p* < 0.05) in these simple screening models were included in a multiple model. The multiple model was run in two versions: 

Model 1a: Including weight status based on BMI derived from measured height and weight as independent variable.

Model 1b: Excluding weight status based on BMI derived from measured height and weight as independent variable.

Model 1b simulates the situation in a study where only parental reports are available. In this situation, it is important to know how parental reports differ from measured values and which variables predict these differences independently of the actual weight status.

Logistic regression models (Model 2) were used to identify potential predictors for the misclassification of weight status. Misclassification was defined as discordance between weight status determined by measured *vs.* parent-reported data. Four different models based on different analysis populations were built:

Model 2a: Includes all overweight individuals (n = 679 boys and n = 615 girls), with the target variable ‘overweight misclassified by parental reports as normal weight or underweight’. The probability for a ‘yes’-response is equal to 1−sensitivity for overweight (n = 214 boys and n = 255 girls misclassified).

Model 2b: Includes all normal weight individuals (n = 3,636 boys and n = 3,561 girls), with the target variable ‘normal weight misclassified by parental reports as overweight’ (n = 224 boys and n = 178 girls misclassified).

Model 2c: Includes all normal weight individuals (n = 3,636 boys and n = 3,561 girls), with the target variable ‘normal weight misclassified by parental reports as underweight’ (n = 587 boys and n = 609 girls misclassified).

Model 2d: Includes all underweight individuals (n = 347 boys and n = 313 girls), with the target variable ‘underweight misclassified by parental reports as normal weight or overweight’. The probability for a ‘yes’-response is equal to 1−sensitivity for underweight (n = 145 boys and n = 118 girls misclassified).

In order to develop a multiple logistic regression model, all potential predictors were initially screened in simple logistic regression models including only one predictor variable at a time (plus age as an adjustment variable). All variables which showed significance (*p* < 0.05) in these simple screening models were entered in the multiple model. Logistic regression models were conducted adjusted for age (as a continuous variable) and separately for boys and girls.

## 3. Results

The proportion of missing parental reports was considerably higher among 11- to 17-year olds (9.7%) than among younger children (3.3%–3.5%; data not shown). The final study sample consisted of 9,187 children and adolescents (4,662 boys and 4,525 girls) aged 2‑17 years (Table 1). About 19.5% of the adolescents felt “too thin”, while 18.5% perceived themselves as “too fat”. In boys, the proportion who felt “too thin” was higher compared to girls, whereas more girls perceived themselves as “too fat” compared to boys. The proportions of under- and overweight based on parental reports differed mostly from those based on measured data. Overall, the proportion of underweight based on parent-reported data was 16.2%, whereas the proportion based on measured values was lower with 7.2% (McNemar *p* < 0.001). For overweight, the overall proportion based on parental and measured reports differed slightly (13.8% *vs.* 14.5%, McNemar *p* = 0.055). 

**Table 1:** Description of the study population.

	**Boys** (n = 4,662)		**Girls** (n = 4,525)		**Total** (n = 9,187)	
	**N**	**%**	**N**	**%**	**N**	**%**
**Age**						
2–6 years	1,467	31.5	1,462	32.3	2,929	31.9
7–10 years	1,275	27.3	1,215	26.9	2,490	27.1
11–17 years	1,920	41.2	1,848	40.8	3,768	41.0
**Weight status (based on measured height and weight)**						
Underweight	347	7.4	313	6.9	660	7.2
Normal weight	3,636	78.0	3,561	78.7	7,197	78.3
Overweight	679	14.6	651	14.4	1,330	14.5
**Weight status (based on parent-reported height and weight)**						
Underweight	732	15.7	756	16.7	1,488	16.2
Normal weight	3,236	69.4	3,191	70.5	6,427	70.0
Overweight	694	14.9	578	12.8	1,272	13.8
**Parents' perception of child’s weight**						
Too thin	1,036	22.7	723	16.3	1,759	19.5
Right weight	2,784	61.0	2,805	63.1	5,589	62.0
Too fat	744	16.3	920	20.7	1,664	18.5
Missing	98		77		175	
**Socio-economic status**						
Low	1,282	27.9	1,212	27.2	2,494	27.6
Medium	2,149	46.8	2,105	47.2	4,254	47.0
High	1,159	25.3	1,144	25.6	2,303	25.4
Missing	72		64		136	
**Migration background**						
Migrant	656	14.1	595	13.2	1,251	13.7
Non migrant	3,994	85.9	3,918	86.8	7,912	86.3
Missing	12		12		24	
**Parental overweight (derived from self-reports of height and weight)**						
Both	1,038	22.7	960	21.6	1,998	22.1
One	2,008	43.9	2,059	46.3	4,067	45.1
None	1,530	33.4	1,431	32.2	2,961	32.8
Missing	86		75		161	

**Figure 1:**
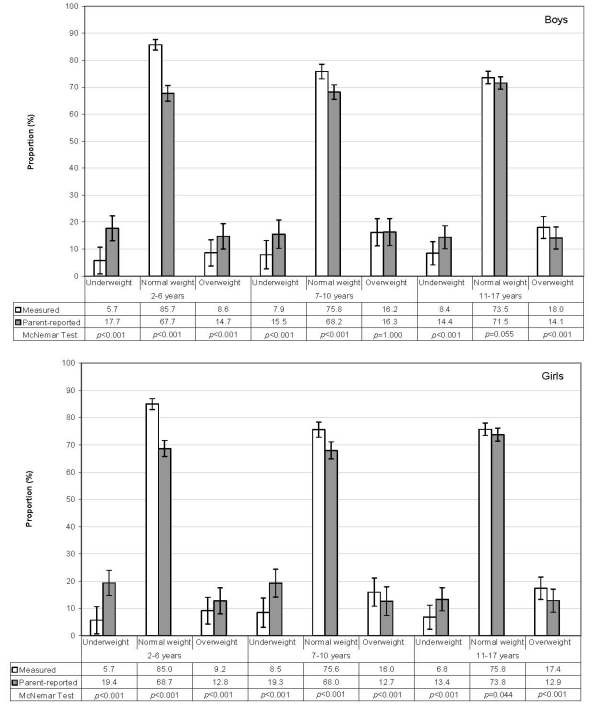
Proportions of underweight, normal weight and overweight by age groups for boys and girls.

Figure 1 illustrates the proportion of weight status by gender and age group. The overestimation of proportions for underweight occurred at all ages (McNemar *p* < 0.001 for all age groups), but the bias was strongest in children aged 2–6 years. The proportion of overweight children was overestimated by parental reports for children aged 2–6 years (McNemar *p* < 0.001), whereas for older children and adolescents, the proportion of overweight was underestimated compared to measured data (McNemar *p* < 0.001), except for boys aged 7–10 years (McNemar *p* = 1.000). For normal weight, the proportions were overestimated, the overestimation getting stronger with increasing age (McNemar *p* < 0.001 for age group 2–6 and 7–10). Proportions for normal weight adolescents aged 11–17 years were quite accurate. For boys, the significance level was borderline (McNemar *p* = 0.55).

Gender- and age group-specific values for sensitivity and specificity are presented in Table 2. The sensitivity for under- and overweight was low (54.8–74.8%). Specificity was higher for overweight (90.2–98.3%) than for underweight (82.7–91.1%). The sensitivity and specificity of parent-reported values had the tendency to get higher with increasing age, with few exceptions. Sensitivity of under and overweight boys aged 7–10 years was higher compared to the other age groups, whereas overweight girls in the age of 7–10 had a lower sensitivity in comparison to the other age groups. Underweight girls aged 11–17 had a higher sensitivity than boys. Sensitivity of overweight boys in the age of 7–10 years was higher in comparison to girls.

**Table 2:** Sensitivity and specificity (in percent) for underweight and overweight by sex and age groups.

	Boys				Girls			
	Sensitivity		Specificity		Sensitivity		Specificity	
	%	95% CI	%	95% CI	%	95% CI	%	95% CI
**Underweight**								
2–6 years	58.3	47.8–68.8	84.8	77.1–92.5	54.8	44.2–65.4	82.7	74.6–90.8
7–10 years	65.3	56.0–74.6	88.8	82.6–95.0	69.9	61.0–78.8	86.2	79.5–92.9
11–17 years	61.1	53.6–68.6	89.9	85.3–94.5	74.6	67.0–82.2	91.1	86.1–96.1
**Overweight**								
2–6 years	66.7	58.5–74.9	90.2	85.0–95.4	67.1	59.2–75.0	91.2	86.4–96.0
7–10 years	72.0	65.9–78.1	94.5	91.4–97.6	59.8	52.9–66.7	96.2	93.5–98.9
11–17 years	67.1	62.1–72.1	97.5	95.9–99.1	65.8	60.6–71.0	98.3	96.9–99.7

Descriptive statistics on measured and parent-reported height, weight, and BMI varied by sex and age group and are illustrated in Table 3. For boys aged 7‑10 and 11‑17 years, height was slightly under-reported, the under-reporting getting stronger with increasing age. For girls aged 2‑6 years, height was slightly over-reported. For boys aged 2‑6 years and girls aged 7‑17 years, height differences between parent-reported and measured values were not statistically different from zero. Weight and BMI were under-reported in all age groups and both sexes, the under-reporting getting stronger with increasing age (*p* < 0.001). Student’s t-test showed a significant difference between the sexes for the difference in BMI derived from parent-reported and measured height and weight in all age groups (2‑6 years: *p* < 0.05; 7‑10 years: *p* < 0.01; 11‑17 years: *p* < 0.001), with a stronger underestimation for girls than for boys. Height differences also differed between boys and girls (2‑6 years: *p* < 0.01; 11‑17 years: *p* < 0.001) except for children aged 7‑10 years (*p* = 0.130). For weight differences, there was no statistically significant difference between boys and girls in any age group (data not shown).

Table 4 presents the results of the multiple linear regressions for BMI difference with the potential predictors age, weight status based on measured data, parental perception of child’s weight, socio-economic status, migration background, and parental overweight (derived from self-reported height and weight). Age was a significant predictor of the BMI difference between parent-reported and measured data (*p* < 0.001) with stronger parental under-reporting with increasing age. The multiple model including measured weight status as independent variable (Model 1a) showed that parents of overweight boys and girls underestimated their children’s BMI more strongly than parents of normal weight children (*p* < 0.001). For underweight children and adolescents, however, the regression coefficient was positive compared to normal weight children (*p* < 0.001). The combined effect of age and parent's perception of their child's weight can be obtained by summing the regression coefficients for intercept, age and parent's perception of child's weight, assuming that all other covariates are at their reference level. 

**Table 3:** Descriptive statistics for measured and parent-reported height, weight and BMI by gender and age groups.

	Boys (n = 4,662)									Girls (n = 4,525)								
	2–6 years			7–10 years			11–17 years			2–6 years			7–10 years			11–17 years		
	Mean	SD	*p ^a^*	Mean	SD	*p ^a^*	Mean	SD	*p ^a^*	Mean	SD	*p ^a^*	Mean	SD	*p ^a^*	Mean	SD	*p ^a^*
**Measured data**																		
Height (cm)	**107.9**	11.03		**136.2**	8.62		**165.7**	13.13		**107.0**	11.48		**135.4**	9.33		**160.8**	8.57	
Weight (kg)	**18.7**	4.38		**32.6**	8.19		**57.8**	16.55		**18.3**	4.63		**32.2**	8.55		**54.6**	12.95	
BMI (kg/m²)	**15.9**	1.59		**17.4**	2.83		**20.7**	3.99		**15.8**	1.59		**17.3**	2.94		**21.0**	3.98	
**Parent-reported data**																		
Height (cm)	**107.9**	11.86		**135.9**	10.38		**164.8**	14.15		**107.4**	12.33		**135.4**	10.70		**160.7**	9.60	
Weight (kg)	**18.5**	4.44		**31.8**	7.84		**55.6**	15.86		**18.0**	4.75		**31.2**	8.25		**52.5**	12.67	
BMI (kg/m²)	**15.8**	2.65		**17.1**	3.12		**20.2**	3.95		**15.5**	2.44		**16.8**	3.25		**20.2**	3.87	
**Individual difference between parent-reported and measured data**																		
Height (cm)	**−0.07**	5.13	0.586	**−0.31**	5.36	0.038	**−0.83**	4.84	0.000	**0.41**	4.73	0.001	**0.03**	5.77	0.874	**−0.06**	3.84	0.486
Weight (kg)	**−0.24**	1.84	0.000	**−0.75**	2.96	0.000	**−2.23**	5.24	0.000	**−0.26**	1.88	0.000	**−1.07**	3.04	0.000	**−2.11**	4.49	0.000
BMI (kg/m²)	**−0.05**	2.26	0.387	**−0.23**	2.08	0.000	**−0.56**	2.13	0.000	**−0.26**	2.08	0.000	**−0.49**	2.24	0.000	**−0.79**	1.93	0.000

^a^ paired samples t-test for difference from zero.

**Table 4:** Summary of multiple linear regression models of BMI difference derived from parent-reported and measured height and weight for boys and girls.

	Boys (n = 4,662)				Girls (n = 4,525)			
	Model 1a		Model 1b		Model 1a		Model 1b	
	B	*p*	B	*p*	B	*p*	B	*p*
**Intercept**	−0.06		−0.05		0.03		0.08	
**Age ^a^**								
2	0.00	ref.	0.00	ref.	0.00	ref.	0.00	ref.
3	0.02	0.925	0.01	0.960	−0.26	0.125	−0.27	0.107
4	−0.10	0.575	−0.10	0.579	−0.18	0.282	−0.18	0.284
5	−0.18	0.295	−0.18	0.314	−0.16	0.354	−0.15	0.369
6	−0.44	0.011	−0.44	0.011	−0.22	0.193	−0.24	0.156
7	0.03	0.863	0.01	0.972	−0.27	0.108	−0.28	0.097
8	−0.30	0.078	−0.29	0.093	−0.30	0.069	−0.28	0.102
9	−0.30	0.075	−0.29	0.094	−0.61	0.000	−0.58	0.000
10	−0.52	0.002	−0.48	0.005	−0.32	0.057	−0.28	0.098
11	−0.42	0.014	−0.38	0.028	−0.29	0.082	−0.26	0.127
12	−0.24	0.168	−0.16	0.368	−0.76	0.000	−0.74	0.000
13	−0.46	0.007	−0.43	0.013	−0.45	0.008	−0.46	0.008
14	−0.71	0.000	−0.69	0.000	−0.55	0.001	−0.51	0.003
15	−0.66	0.000	−0.68	0.000	−0.72	0.000	−0.68	0.000
16	−0.75	0.000	−0.76	0.000	−0.81	0.000	−0.78	0.000
17	−0.79	0.000	−0.78	0.000	−0.73	0.000	−0.67	0.000
**Weight status (based on measured height and weight)**								
Underweight	0.55	0.000	not included in the model		0.66	0.000	not included in the model	
Overweight	−0.67	0.000			−0.78	0.000		
Normal weight	0.00	ref.			0.00	ref.		
**Parents’ perception of child’s weight**								
Too thin	0.28	0.001	0.43	0.000	0.13	0.156	0.34	0.000
Too fat	−0.17	0.139	−0.61	0.000	−0.18	0.070	−0.63	0.000
Right weight	0.00	ref.	0.00	ref.	0.00	ref.	0.00	ref.
**Socio-economic status**								
Low	0.23	0.010	0.18	0.041	0.01	0.937	−0.06	0.456
Medium	−0.01	0.920	−0.02	0.792	−0.11	0.133	−0.14	0.058
High	0.00	ref.	0.00	ref.	0.00	ref.	0.00	ref.
**Migration background**								
Migrant	0.37	0.000	0.33	0.001	0.29	0.002	0.27	0.005
Non-Migrant	0.00	ref.	0.00	ref.	0.00	ref.	0.00	ref.
**Parental overweight (derived from self-reports of height and weight)**								
Both	−0.07	0.438	−0.13	0.122	−0.03	0.768	−0.11	0.218
One	0.04	0.614	0.01	0.842	−0.16	0.022	−0.18	0.009
None	0.00	ref.	0.00	ref.	0.00	ref.	0.00	ref.
**R²**	0.051		0.041		0.055		0.040	
**F-statistics**	11.20 ***		9.87 ***		11.70 ***		9.34 ***	

^a^ In years, as categorical variable. *B =* regression coefficient; *p = p*-value; ref. = reference. ***p < 0.001. Model 1a: Multiple model with measured weight status. Model 1b: Multiple model without measured weight status.

These calculations show that BMI derived from parent-reported height and weight was overestimated or reported with almost no bias in underweight children up to age 13 in boys and 15 in girls, and underestimated to a lower extent compared to normal weight individuals for adolescents older than this. For boys, BMI derived from parental reports was underestimated less by parents perceived them to be ‘too thin’ (*p* = 0.001) instead of having ‘the right weight’, which results in an overestimation of BMI based on parental reports for underweight boys considered ‘too thin’ up to age 13 (and a more or less unbiased reporting for older boys), while the BMI of normal weight boys considered ‘too thin’ is still underestimated by parental reports at most ages (except for 2- to 4- and 7-year olds). BMI derived from parental reports was borderline more strongly underestimated for girls if they were perceived as ‘too fat’ (*p* = 0.07) in comparison to being regarded as having the ‘right weight’.

Parents with a low socio-economic status underestimated the BMI derived from parent-reported height and weight of their boys to a lower extent (*p* = 0.01) than parents with a high socio-economic status, while socio-economic status showed no effect on the difference in BMI in the multiple model for girls. For girls who had one overweight parent, BMI based on parental reports was more strongly underestimated compared to girls who had no overweight parent, whereas parental overweight had no effect in the model for boys. BMI derived from parental reports showed less underestimating in parents with a migration background (*p* < 0.001) compared to non-migrated parents. The model not including weight status (Model 1b) showed similar results with the exception of the predictor ‘parental perception of child’s weight’, which showed stronger effects than in Model 1a. 

The multiple logistic regression Model 2a is displayed in Table 5. Out of 679 overweight boys and 651 overweight girls, 214 boys (31.5%) and 255 girls (41.5%) were misclassified by parental reports as normal weight or underweight. Model 2a showed that overweight boys and girls who are considered as ‘too fat’ by their parents had lower odds to be misclassified as normal- or underweight (*p* < 0.001) than participants who were seen to have the ‘right weight’. Parents with a low socio-economic status had lower odds to misclassify their overweight girls, but not boys, as normal weight or underweight (*p* < 0.001) compared to parents with a high socio-economic status. In Model 2b, the misclassification of normal weight individuals (n = 3,636 boys and n = 3,561 girls) as overweight by parental reports of height and weight is described. The percentage of misclassified children is lower than in Model 2a, namely 224 boys (6.2%) and n = 178 girls (5.0%). Normal weight children who are perceived as ‘too fat’ by their parents had higher odds to be misclassified as overweight (OR = 3.5, 95% CI 2.2–5.6 for boys and OR = 3.0, 95% CI 1.9–4.6 for girls) compared to boys and girls who are perceived by their parents as having the ‘right weight’. Parents with a low socio-economic status had higher odds to misclassify their normal weight children as overweight (OR = 1.9, 95% CI 1.3–2.8 for boys and OR = 1.7, 95% CI 1.1–2.6 for girls) in comparison to parents with a high socio-economic status.

Parents with a migration background had higher odds to misclassify their children as overweight (OR = 2.0, 95% CI 1.4–2.8 for boys and OR = 1.7, 95% CI 1.1–2.6 for girls) compared to non-migrants. If both parents are overweight, there were higher odds that they misclassify their normal weight children as overweight (OR = 1.5, 95% CI 1.04–2.3 for boys and OR = 1.9, 95% CI 1.2–2.9 for girls) compared to non-overweight parents. For boys this effect was even seen if just one parent was overweight (OR = 1.4, 95% CI 1.01–2.0). Model 2c looks at the misclassification of normal weight as underweight. Out of 3,636 normal weight boys and 3,561 normal weight girls, 587 boys (16.1%) and 609 girls (17.1%) were misclassified as underweight. Normal weight children who are considered as ‘too thin’ by their parents had higher odds to be misclassified as underweight (OR = 2.5, 95% CI 2.1–3.1 for boys and OR = 3.0, 95% CI 2.4–3.8 for girls) compared to children who are perceived as having the ‘right weight’. If they are regarded as ‘too fat’, the odds of being misclassified as underweight through parental reports were lower (OR = 0.3, 95% CI 0.2–0.6 for boys and OR = 0.2, 95% CI 0.1–0.4 for girls) in comparison to participants who are regarded as having the ‘right weight’. Model 2d analyses the misclassification of underweight children as normal weight or overweight. Out of 347 underweight boys and 313 underweight girls, 145 boys (41.8%) and 118 girls (37.7%) were misclassified as normal weight or overweight by parental reports of height and weight. The model demonstrated that parents of underweight boys, but not girls, had lower odds to misclassify their children as normal weight or overweight if they perceived them to be ‘too thin’ (OR = 0.5, 95% CI 0.3–0.8) compared to boys who are perceived as ‘right weight’ (for brevity, data for Model 2b–2d are not shown).

**Table 5:** Multiple logistic regression models for overweight boys and girls misclassified by parental reports as normal weight or underweight.

	Model 2a ^a^					
	Boys (n = 679)			Girls (n = 651)		
	OR	95% CI	*p*	OR	95% CI	*p*
**Intercept**	0.72			3.86		
**Age**^b^						
	1.05	1.01–1.10	0.014	1.01	0.96–1.05	0.748
**Parents’ perception of child’s weight**						
Too thin ^c^	-	-	-	-	-	-
Too fat	0.26	0.17–0.39	0.000	0.22	0.13–0.36	0.000
Right weight	1.00	ref.	ref.	1.00	ref.	ref.
**Socio-economic status**						
Low				0.39	0.23–0.65	0.000
Medium	not included in the multiple model ^d^			0.66	0.40–1.07	0.094
High				1.00	ref.	ref.
**R²**	0.098			0.119		
**Log-Likelihood**	777.48			796.38		
**Chi-squared**	48.16 ***			58.87 ***		

^a^ Migration background and parental overweight showed no significance in simple analyses and are thus not included in the table. ^b^ In years, as continuous variable. ^c^ No overweight participant who was classified by parental reports as normal weight or underweight was perceived as ‘too thin’. ^d^ since the variable was not significant in the simple logistic regression model. *** *p* < 0.001. OR = odds ratio; *p = p*-value; ref. = reference. *Model 2a:* Includes all overweight individuals, with the target variable ‘overweight misclassified by parental reports as normal weight or underweight’ (n = 214 boys and n = 255 girls misclassified).

## 4. Discussion

The present study indicates that the main predictors of the difference between parent-reported and measured values are age, gender, weight status and parents’ perception of their child’s weight. Socio-economic status, migration background and parental overweight only show an effect in some of the analyses.

Height differences vary by gender, but are generally small. Weight and BMI derived from parental reports are underestimated in all age groups and both sexes, the underestimation getting stronger with increasing age. An explanation might be that parents are not up-to-date with the current measurements of height and weight of their older children compared to the younger ones. This is also seen by the higher proportion of missing values in older individuals. Weight and height in younger children are checked regularly, e.g., in screening examinations. The interval between screening examinations is getting larger with increasing age. A further explanation for the smaller BMI difference in 2- to 6-year-olds might be the adiposity rebound [25]. Whereas BMI percentiles continuously increase with age for school-aged children and adolescents, so that parental reports of an outdated measurement will underestimate the current BMI of their child in this age group, the situation is different for pre-school children around the age of the adiposity rebound. BMI percentiles in 2- to 6-year-olds first show a decrease, followed by an increase after the adiposity rebound, so that reporting an outdated measurement will not lead to a systematic under-reporting. Finally, as the range of height, weight and BMI values increases with age, a misreporting at higher ages becomes more possible. However, the differences between the age groups remain when differences between parent-reported and measured data are expressed as percentages of the measured values.

In the entire study population, proportions for overweight derived from parent-reported height and weight are quite accurate, with a tendency towards an underestimation of the measured prevalence, contradictory to the results of Akinbami and Ogden [16] who saw an overestimation of overweight. However, as already mentioned in the introduction, the study of Akinbami and Ogden compared two separate national U.S. surveys and was therefore unable to compare BMI estimates for individual children. The results by age groups are similar to those seen by Akinbami and Ogden [16]: Overweight in younger individuals is overestimated, whereas for older ones overweight is underestimated. Underestimation of overweight in children and adolescents aged 7–17 years is primarily driven by an under-reporting of weight through parents’ reports. Underweight is overestimated in all age groups as seen by Akerman *et al.* [17], in our study the bias being strongest in the group of 2–6 year-olds.

The linear regression models with the difference in BMI based on parent-reported *vs.* measured height and weight as target variable show that the actual weight status has a large effect on the possible bias of the parent reports. While parents of overweight children underestimate their child’s BMI derived from parent-reported height and weight to a stronger extent than parents of normal weight children, the effect is in the opposite direction for underweight children and adolescents, as found in other studies [15,17]. Model 1b simulates the situation that only parental reports are available and indicates that the parents’ perception of their child’s weight could be used to approximate the actual weight status and thus supplement parent-reported height and weight in the development of a correction formula. The logistic regression analysis of the misclassification probabilities underlines the importance of parents’ perception of child’s weight by showing, for example, that overweight adolescents who are perceived as ‘too fat’ were less often misclassified as normal weight or underweight. This could be a hint that the underestimation of the weight status of overweight boys and girls occurs if their parents are not aware of the overweight and perceive their children as of the ‘right weight’. 

Low socio-economic status and migration background have an effect on the BMI difference derived from parent-reported *vs.* measured height and weight and on the probability of misclassification in some of the models. This may be related to the fact that children from low-income families and with migration background are heavier [26] and if these children are near the cut off between normal weight and overweight, then a misclassification will be more likely, even if the difference between BMI based on parent-reported *vs.* measured values is small.

This study has strengths and limitations. Major strengths of the KiGGS study are the large sample size and the wide age range. Further strengths are the fact that height and weight were measured in a standardized way and that the collection of parent-reported and measured data took place at the same time. Thus, this study provides individual differences for parent-reported and measured height, weight and BMI. Another advantage is the high number of covariables that could be included in the regression models. A limitation of this study is that parents might have read the description of the study procedures, which had been available in the internet beforehand, and thus may have been aware that height and weight would be measured following the parental reports. Furthermore, it is not known whether the parents had measured height and weight of their children and adolescents recently prior to the survey, so it is unclear which age the parent-reported measurements in fact correspond to. In principle, the difference in accuracy between self-reports (to the nearest cm) and measurements (to the nearest mm) may be a further limitation, but a sensitivity analysis with rounded measured data led to nearly the same results. Another weakness is that the analysis is restricted to children and adolescents aged 2 years or older because of the limited validity of the national German BMI reference below this age range [20]. In a sensitivity analysis for children aged 0–2 years, over-reporting of underweight and overweight was stronger than in the group of 2- to 6-year-olds. Also, values for sensitivity and specificity were lower compared to the other age groups.

## 5. Conclusions

In conclusion, the results of this study do not support the exclusive use of parent-reported height and weight for assessment of prevalence rates of weight status. The parental perception of their child’s weight emerged as an important predictor of the accuracy of parent-reported height and weight. Thus, the collection of this information is recommended in addition to parental reports. Further research is necessary to develop a correction formula that generates more accurate data than using uncorrected parental reports.
